# Rebound Pruritus and Urticaria Post-discontinuation of Chronic Cetirizine Use: A Case Report

**DOI:** 10.7759/cureus.100214

**Published:** 2025-12-27

**Authors:** Jun Jie Benjamin Seng, Meijin Cai, Prawira Oka

**Affiliations:** 1 Family Medicine, SingHealth Polyclinics, Singapore, SGP

**Keywords:** adverse drug reaction, antihistamines, antihistamine withdrawal, post-discontinuation pruritus, rebound pruritus

## Abstract

Cetirizine is a common over-the-counter antihistamine used to treat allergic rhinitis, eczema, and urticaria. There have been increasing reports showing increased risk of rebound pruritus following discontinuation of long-term antihistamine use in the United States and the Netherlands. However, evidence on this condition and its management among Asian populations remains limited. We report the case of a Chinese male in his 50s with a background of hypertension, hyperlipidaemia, and chronic urticaria who had been regularly taking over-the-counter cetirizine for the past two years. He presented with recurrent urticaria, characterized by wheals and severe pruritus over his forearms, emerging two to three days after each cetirizine discontinuation attempt. A diagnosis of rebound pruritus and urticaria post-discontinuation of cetirizine was made. Through shared decision-making, he was switched to loratadine and chlorpheniramine for three days before transitioning to an “as-needed” regimen without recurrence of pruritus or urticaria. While the pathophysiology of rebound pruritus and urticaria post-discontinuation of chronic cetirizine use remains poorly understood, this case report suggests that the phenomenon may be drug-specific rather than a class-specific adverse effect. Larger studies are necessary to confirm if a temporary switch and short-term bridging with alternative antihistamines offer a safer discontinuation strategy for patients on chronic cetirizine.

## Introduction

Cetirizine is a second-generation antihistamine that works by competitively and selectively inhibiting H1-receptors in the respiratory and gastrointestinal systems [1]. Owing to its poor penetration across the blood-brain barrier, it has minimal sedating properties compared to first-generation antihistamines [2]. Consequently, in conditions such as chronic idiopathic urticaria, atopic dermatitis, and allergic rhinitis, cetirizine is commonly used long-term due to its established efficacy and favourable safety profile [3].

Pruritus and urticarial reactions following discontinuation of chronic cetirizine use were first highlighted in patient forums and blogs [4,5]. In May 2025, the United States Food and Drug Administration (FDA) issued a warning regarding this rare potential complication of severe pruritus following the discontinuation of cetirizine and its enantiomer, levocetirizine [6]. A review of the FDA Adverse Event Reporting System (FAERS) database identified 146 cases of pruritus following cetirizine discontinuation [7]. Among patients who underwent a re-challenge with cetirizine, nearly all experienced recurrence of pruritus (n = 54/55, 98.1%) [7]. Similarly, data from the Netherlands Pharmacovigilance Centre Lareb reported 12 cases of patients experiencing severe pruritus following cessation of chronic cetirizine therapy [5].

Currently, there is limited literature on rebound pruritus among Asian patients. In addition, while strategies such as gradual tapering of cetirizine, switching to an alternative antihistamine, or reinitiating cetirizine have been proposed, evidence on the optimal discontinuation strategy remains limited [6]. 

## Case presentation

This case was reported according to the CARE 2013 checklist [8]. A Chinese male in his early 50s with a body mass index of 24.5 kg/m^2^ presented to the outpatient clinic in July 2025 for his six-monthly chronic disease consultation. He had a significant medical history of hypertension, hyperlipidemia, chronic idiopathic urticaria, and prior vitamin B12 and iron deficiency anemia. He had no past surgical history and no known drug or food allergies.

With regard to medications, he was on telmisartan 40 mg every morning, nifedipine (long-acting) 60 mg every morning, ferrous gluconate one capsule once daily, and mecobalamin 500 mcg once daily. In addition, he had been taking over-the-counter cetirizine 10 mg daily for the past two years for his chronic urticaria. There had been no medication changes for the past year. As his urticaria was well controlled, he was keen to discontinue cetirizine. However, he reported recurrent urticaria post-discontinuation of cetirizine on two separate occasions in the past year. The urticaria appeared two to three days after stopping cetirizine, presenting as non-painful wheals over his bilateral forearms and trunk. Re-initiation of cetirizine promptly led to the resolution of symptoms within two to three days. He remained well during each episode and denied any intercurrent illness, new food or medications, or exposure to dusty environments. At the initial consultation, his urticaria was quiescent, and physical examination was unremarkable. 

With regard to biochemical parameters, the patient’s latest renal function, liver function, full blood count test, vitamin B12, folate, and iron indices were within normal range (Table 1). Details related to his metabolic profile are also reported in Table 1.

**Table 1:** Patient's baseline biochemical and hematological profile

Laboratory parameters (units)	Patient’s reading	Reference range
Renal		
Sodium (mmol/L)	142	136–146
Potassium (mmol/L)	4.3	3.6–5.0
Chloride (mmol/L)	101	100–107
Creatinine (mmol/L)	85	54–85
Estimated glomerular filtration rate (mL/min/1.73 m²)	90	Not applicable
Liver		
Alanine transaminase (U/L)	25	6–66
Metabolic profile		
Total cholesterol (mmol/L)	6.01	<5.2
High density lipoprotein (mmol/L)	1.19	≥1.0
Triglycerides (mmol/L)	2.15	<1.7
Low density lipoprotein (mmol/L)	3.84	≤4.1
Fasting glucose (mmol/L)	4.9	3.9–6.0
Haematological		
Haemoglobin (g/dL)	15.6	12.0–16.0
White blood count (/L)	8.5	4–10 x 10^9^
Platelet count (/L)	440	140–440 x 10^9^
Vitamin B12 (pmol/L)	242	145–637
Folate (nmol/L)	>50.5	>13.4
Transferrin saturation (%)	43.9	16–55
Ferritin (µg/L)	47	47.0–452

**Figure 1:**
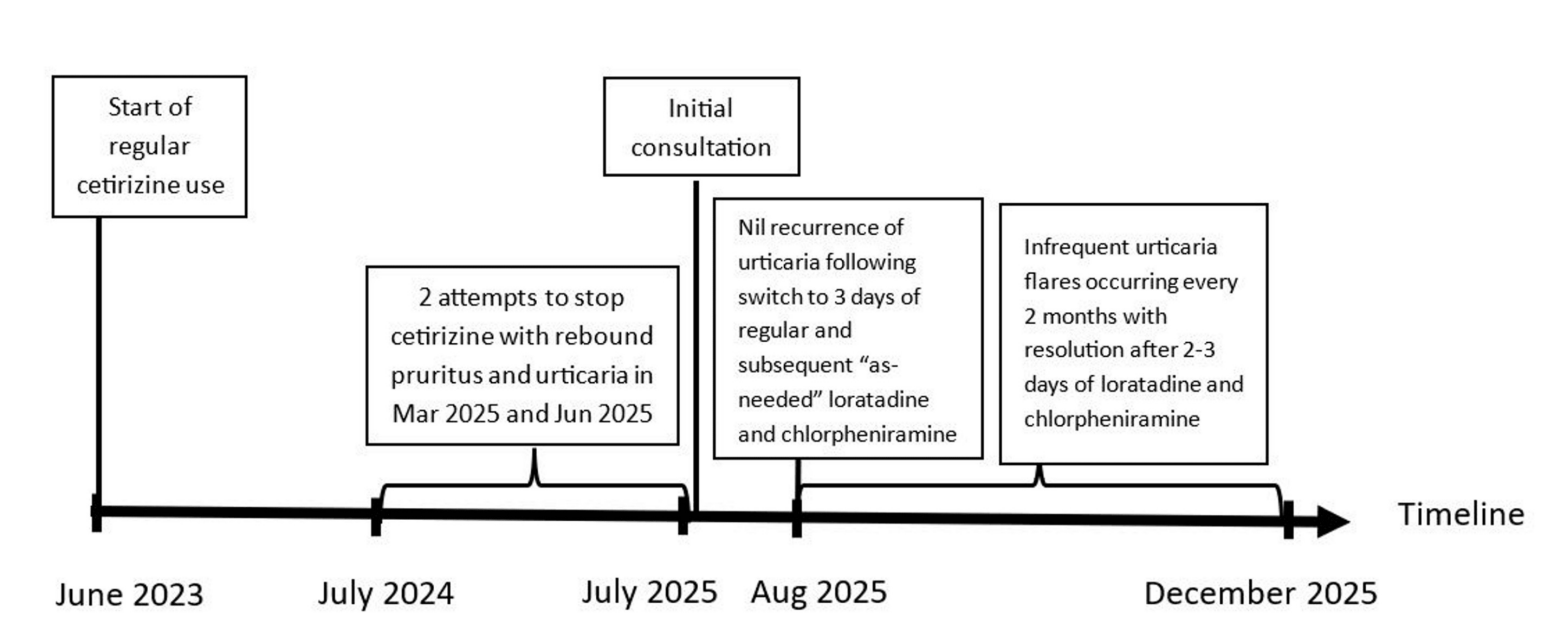
Timeline of events

The timeline of events is shown in Figure 1.

Investigations

Nil investigations were performed as the patient was asymptomatic at the time of evaluation.

Differential diagnosis

The primary working diagnosis was rebound pruritus and urticaria following the discontinuation of chronic cetirizine use. The diagnosis was supported by a modified Naranjo adverse drug reaction probability scale score of 7 (probable) (Table 2) [9]. Another differential considered was a potential urticaria flare.

**Table 2:** Modified Naranjo adverse drug reaction probability scale [9]* *Modification to questions 2-4 and 9 were made in view of the adverse drug reaction occurring post-discontinuation of cetirizine use rather than during cetirizine use. The table is published under Creative Commons License.

Question	Yes	No	Do not know	Score
1. Are there previous conclusive reports on this reaction?	+1	0	0	+1
2. Did the adverse event appear after the suspected drug was discontinued?	+2	-1	0	+2
3. Did the adverse event improve when the drug was readministered or a specific antagonist was administered?	+1	0	0	+1
4. Did the adverse event reappear when the drug was discontinued?	+2	-1	0	+2
5. Are there alternative causes that could on their own have caused the reaction?	-1	+2	0	-1
6. Did the reaction reappear when a placebo was given?	-1	+1	0	0
7. Was the drug detected in blood or other fluids in concentrations known to be toxic?	+1	0	0	0
8. Was the reaction more severe when the dose was increased or less severe when the dose was decreased?	+1	0	0	0
9. Did the patient have a similar reaction to the same or similar drugs in any previous discontinuation?	+1	0	0	+1
10. Was the adverse event confirmed by any objective evidence?	+1	0	0	+1
Total score	7			

Treatment

Shared decision-making with the patient considered the recurrent episodes of rebound urticaria following cetirizine discontinuation and his preference to be on “as-needed” antihistamines for chronic idiopathic urticaria. He was switched to loratadine 10 mg every morning and chlorpheniramine 4 mg every night for three days with subsequent conversion to “as-needed” use. The three-day bridging regime accounted for cetirizine’s half-life of up to 12 hours, ensuring sufficient time for the medication to wash out. Subsequently, therapy was transitioned to an “as-needed” regimen in line with the patient’s preferences.

Outcome and follow-up

There was no recurrence of pruritus or urticaria in the four weeks following the switch and eventual transition to “as-needed” antihistamine use. Subsequently, the patient experienced infrequent episodes of urticaria every two months that resolved with a two to three-day course of loratadine and chlorpheniramine.

## Discussion

To the best of our knowledge, this is the first case of rebound pruritus and urticaria following the discontinuation of chronic cetirizine use in Singapore, as confirmed with the Health Sciences Authority of Singapore. Overall, the chronological presentation of rebound pruritus and urticaria post-cessation of cetirizine in this patient was similar to other cases reported in the Netherlands and the United States, where the time to pruritus was between one to three days [5,7]. As observed in other published cases, this patient reported symptom resolution post-reinitiation of cetirizine and recurrence with discontinuation [7]. The unique scenario in our patient’s case was a temporary switch to a short course of alternative antihistamines followed by a transition to “as-needed” therapy thereafter.

Although the pathophysiology of the phenomenon remains unclear, potential theories include the downregulation of H1 receptor gene expression with chronic cetirizine use [5]. Hence, the withdrawal of cetirizine following chronic use may result in an increased proportion of receptors being occupied by histamine molecules, culminating in pruritus [5].

In our patient, a successful switch to “as-needed” therapy following a short course of loratadine and chlorpheniramine may have worked due to the differing affinities towards peripheral histamine receptors of the agents. In comparison to cetirizine, both chlorpheniramine and loratadine exhibit reduced affinity to peripheral histamine receptors in in vivo trials [10]. Collectively, the short course of these lower potency antihistamines may have attenuated the impact of cetirizine withdrawal on the H1 receptors, thereby reducing the likelihood of rebound pruritus.

Currently, the optimal management of rebound pruritus following the discontinuation of chronic cetirizine use remains unclear. Proposed strategies include cetirizine re-initiation, cetirizine tapering, alternative antihistamine use, short-term oral prednisolone, and topical steroids [5, 7]. Among these strategies, rates of symptom resolution are only reported for patients who re-initiated cetirizine (75-98%) and underwent cetirizine tapering (5%) [5, 7]. Consequently, the manufacturers of cetirizine have recommended re-initiating cetirizine in patients experiencing rebound pruritus [11]. However, it is unclear if continued cetirizine use could further perpetuate or even increase the likelihood of more severe pruritus following future discontinuation attempts.

From the literature, cetirizine and levocetirizine appear to be the main agents implicated in this adverse reaction. The absence of pruritus following the switch to other antihistamines in our patient suggests that this may be drug-specific rather than a class-specific adverse effect.

There were a few limitations in this case report. First, episodes of cetirizine withdrawal, drug re-challenges, and associated reactions were based on the patient’s recollection, as he did not consult a physician during the episodes. Second, while the Naranjo adverse drug reaction probability scale score for this patient is relatively high, the instrument has not been validated for patients experiencing adverse effects from medication discontinuation. Nonetheless, given the relevance of the items in assessing the likelihood of causality for the adverse reaction, this case report supports future research to validate a modified version of the tool for patients suffering from adverse reactions following medication discontinuation.

## Conclusions

While the pathophysiology of rebound pruritus and urticaria post-discontinuation of chronic cetirizine use remains poorly understood, this case report suggests that the phenomenon may be drug-specific rather than a class-specific adverse effect. A temporary switch to alternative antihistamines may be considered for patients on long-term cetirizine who wish to transition to “as-needed” use, to minimize the risk of rebound pruritus or urticaria. Larger studies are necessary to confirm if a temporary switch and short-term bridging with alternative antihistamines offer a safer discontinuation strategy for patients on chronic cetirizine.
